# The influenza A virus genome packaging network — complex, flexible and yet unsolved

**DOI:** 10.1093/nar/gkac688

**Published:** 2022-08-22

**Authors:** Celia Jakob, Rithu Paul-Stansilaus, Martin Schwemmle, Roland Marquet, Hardin Bolte

**Affiliations:** Institute of Virology, Medical Center – University of Freiburg, 79104 Freiburg, Germany; Faculty of Medicine, University of Freiburg, 79110 Freiburg, Germany; Université de Strasbourg, CNRS, Architecture et Réactivité de l'ARN, UPR 9002, IBMC, 67000 Strasbourg, France; Institute of Virology, Medical Center – University of Freiburg, 79104 Freiburg, Germany; Faculty of Medicine, University of Freiburg, 79110 Freiburg, Germany; Université de Strasbourg, CNRS, Architecture et Réactivité de l'ARN, UPR 9002, IBMC, 67000 Strasbourg, France; Institute of Virology, Medical Center – University of Freiburg, 79104 Freiburg, Germany; Faculty of Medicine, University of Freiburg, 79110 Freiburg, Germany

## Abstract

The genome of influenza A virus (IAV) consists of eight unique viral RNA segments. This genome organization allows genetic reassortment between co-infecting IAV strains, whereby new IAVs with altered genome segment compositions emerge. While it is known that reassortment events can create pandemic IAVs, it remains impossible to anticipate reassortment outcomes with pandemic prospects. Recent research indicates that reassortment is promoted by a viral genome packaging mechanism that delivers the eight genome segments as a supramolecular complex into the virus particle. This finding holds promise of predicting pandemic IAVs by understanding the intermolecular interactions governing this genome packaging mechanism. Here, we critically review the prevailing mechanistic model postulating that IAV genome packaging is orchestrated by a network of intersegmental RNA–RNA interactions. Although we find supporting evidence, including segment-specific packaging signals and experimentally proposed RNA–RNA interaction networks, this mechanistic model remains debatable due to a current shortage of functionally validated intersegmental RNA–RNA interactions. We speculate that identifying such functional intersegmental RNA–RNA contacts might be hampered by limitations of the utilized probing techniques and the inherent complexity of the genome packaging mechanism. Nevertheless, we anticipate that improved probing strategies combined with a mutagenesis-based validation could facilitate their discovery.

## INTRODUCTION

The natural reservoir of influenza A viruses (IAVs) are aquatic birds, yet spill-over events have led to the introduction and subsequent establishment of numerous IAV lineages in other species, including swine, poultry, bats and humans (1,2). The ability of IAVs to cross inter-species barriers and adapt to new hosts is enabled by an enormous genetic variability which is brought about by reassortment events of the eight genome segments between co-infecting IAVs. Genetic reassortment has a great impact on IAV evolution and can create devastating pandemic IAVs, as exemplified by the 1957 (Asian flu), 1968 (Hong Kong flu) and 2009 (Swine flu) pandemic IAVs that originated via reassortment between avian, swine and human virus strains (3–5). As the frequency of reassortment events between avian and mammalian IAVs increases in porcine facilities (6,7), there is growing concern that novel reassortants could invade the human population and elicit the next flu pandemic (2). Thus, a profound understanding of the mechanisms underlying reassortment is urgently needed to predict and combat future pandemic IAVs.

Recent research findings suggest that reassortment is driven by a selective genome packaging mechanism that assembles the eight viral genome segments as a supramolecular complex into the virus particle, thereby allowing the exchange of cognate genome segments between co-infecting IAVs (8–10). The currently favoured mechanistic model postulates that this genome complex forms by means of a network of intersegmental RNA–RNA interactions. In this review, we will scrutinize this mechanistic model by critically reviewing the available body of evidence. We will describe the genome segment loci known to coordinate IAV genome packaging and discuss the significance of recently proposed RNA–RNA interaction networks obtained by high-throughput crosslinking experiments. Although we reveal ample evidence to support the prevailing mechanistic model, we also realize that the identification of functionally relevant RNA–RNA interactions between genome segments remains a major challenge. We would therefore like to stimulate critical rethinking of the experimental approaches used to study intersegmental RNA–RNA interactions and suggest potential avenues to identify functional intermolecular contacts involved in genome packaging and reassortment.

## THE IAV GENOME PACKAGING MODEL AND ITS CHALLENGES

### From single vRNPs to a supramolecular complex

**Figure 1.**
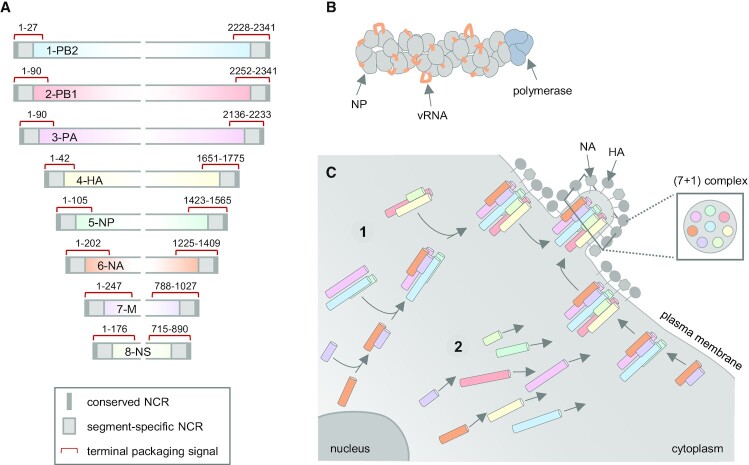
IAV genome structure and genome packaging model. (**A**) An illustration of the IAV genome based on the WSN/H1N1 strain. The genome segments are shown in negative-sense orientation from 3′ to 5′. The identified terminal packaging signals are indicated with red lines. A detailed summary of all characterized packaging signals is presented in (8,56). The conserved 5′ and 3′ segment termini of the non-coding regions (NCRs) are highlighted in dark grey, whereas the segment-specific parts of the NCRs are indicated in light grey. PB, polymerase basic subunit; PA, polymerase acidic subunit; HA, hemagglutinin; NP, nucleoprotein; NA, neuraminidase; M, matrix; NS, non-structural. (**B**) An illustration of a viral ribonucleoprotein (vRNP). (**C**) The current genome packaging model proposes that the eight genome segments sequentially assemble into a (7 + 1) genome complex. This process might either occur (1) *en route* to the plasma membrane or (2) directly at the viral budding site, while the first scenario is currently preferred. The inset depicts a schematic cross-section through a budding virion showing the (7 + 1) arrangement of the vRNPs as observed by electron microscopy (34–36).

The IAV genome comprises eight negative-sense, single-stranded viral RNA segments (vRNAs) that vary in length from 890 to 2341 nucleotides (11). All genome segments share the same structure, characterized by a broad central coding region flanked by two shorter non-coding regions (NCRs). The NCRs consist of conserved terminal regions (spanning 12 nucleotides at the 3′ end and 13 nucleotides at the 5′ end) and segment-specific portions that vary in length between 5 and 45 nucleotides (11–13) (Figure 1A). The conserved NCR termini and two adjacent nucleotides together form a panhandle structure that is bound by the heterotrimeric viral polymerase and serves as a promoter during genome replication (14–18). The remaining portions of the NCRs and the coding regions of the vRNAs are non-uniformly associated with multiple copies of the viral nucleoprotein (NP) (19–21), forming rod-shaped, helical viral ribonucleoproteins (vRNPs) (Figure 1B) (22–26).

Following viral infection, these vRNPs are released from the infecting virus particles into the cytoplasm and imported into the nucleus to be transcribed and replicated. Newly formed vRNPs are then exported out of the nucleus and transported to viral budding sites at the plasma membrane. The prevailing model assumes that during this transport, individual vRNPs are progressively assembled into an octameric supramolecular genome complex (Figure 1C) (27–33). While the specific cellular compartment hosting this assembly process remains unknown, (7 + 1) genome complexes in which seven vRNPs surround a central one are incorporated into viral particles at the plasma membrane (34–37). Throughout this review, these processes of genome complex formation and its subsequent incorporation into virions will be collectively referred to as genome packaging.

Past research suggests that IAV genome packaging is coordinated by a network of specific intersegmental RNA–RNA interactions that is formed by discrete genomic loci known as packaging signals. While initial studies have mapped these packaging signals towards the segment termini, more recent studies suggest that they are also present in internal vRNA regions lying beyond the vRNA termini. In the following sections, we review the current knowledge on packaging signals and revisit their presumed role in forming intersegmental RNA–RNA interactions.

### A network of terminal packaging signals coordinates genome packaging

Terminal packaging signals were first proposed in studies analysing the genome content of defective interfering (DI) IAV particles. Unlike ‘standard’ virus particles which package eight full-length vRNAs, DI particles commonly package one genome segment that harbours a large internal deletion but retains the NCRs plus short portions of the adjacent coding regions (38,39). Such DI-RNAs have been described for all genome segments (40–43), yet often derive from vRNAs 1, 2 and 3 encoding viral polymerase subunits. In these cases, the truncated genomes do not give rise to a functional viral polymerase, which renders the DI particle replication-incompetent. However, during co-infection with infectious ‘standard’ virions when all viral proteins are expressed, these DI-RNAs are replicated and packaged into viral particles. Since DI-RNAs interfere with their full-length counterparts for replication and packaging under co-infection conditions, they can reduce the production of replication-competent ‘standard’ particles (44,45), a finding which coined their nomenclature. These early observations suggested that the retained terminal ends in DI-RNAs mediate vRNA incorporation into virions and thus contain packaging signals.

**Figure 2.**
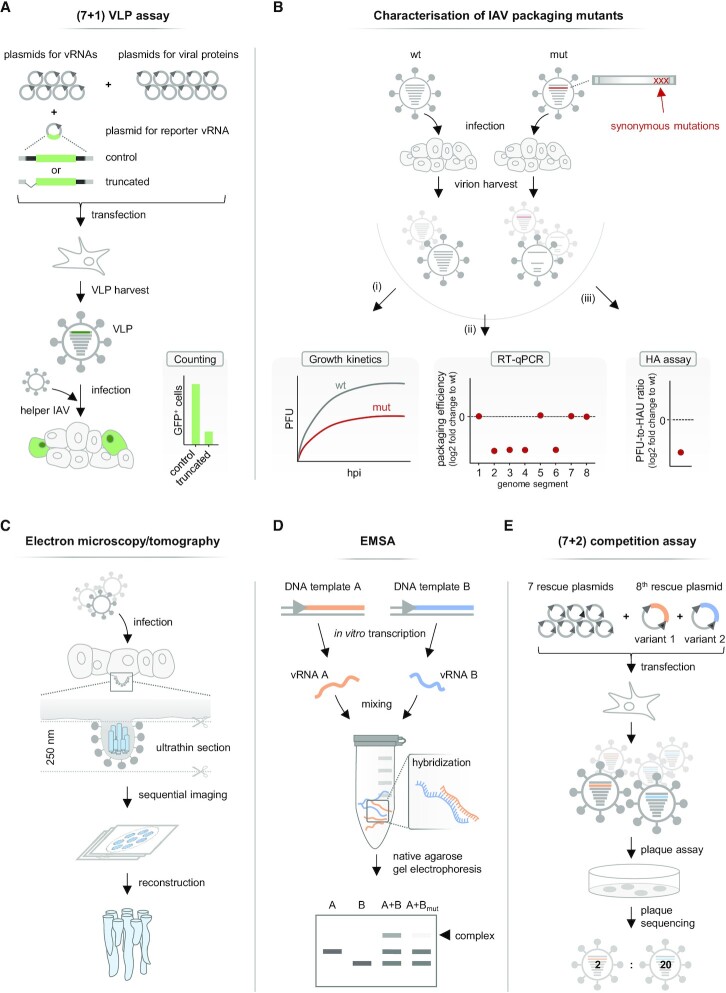
Established methods to study IAV genome packaging. (**A**) (7 + 1) virus like particle (VLP) assay. To generate VLPs, cells are co-transfected with ten plasmids encoding viral proteins, seven plasmids encoding full-length vRNAs and one plasmid encoding an artificial eighth vRNA which comprises a GFP-reporter gene flanked by the 5′ and 3′ ends including the non-coding regions (shown in grey) and parts of the coding region (shown in black) of the vRNA under study. VLPs released from transfected cells are subsequently used to infect new cells. Replication of the reporter vRNA is facilitated by superinfection with a helper IAV. The number of cells expressing GFP reflects the packaging efficiency of the reporter vRNA. Terminal packaging signals were mapped by shortening the coding region as indicated here by a truncated artificial vRNA. Further mappings were performed by introducing synonymous mutations into the terminal packaging signals (not shown). Variations of this (7 + 1) VLP assay are extensively described in (111). (**B**) Characterization of IAV packaging mutants. Cell cultures are infected either with wild-type virus (wt) or a mutant virus with synonymous mutations in a terminal packaging signal (mut). Newly formed viral particles are collected at various time points post-infection (hpi). Viral growth is monitored by determining the number of plaque-forming units (PFU) (i). A decrease of infectious particles can indicate impaired genome packaging. The vRNA amounts packaged into viral particles are measured using RT-qPCR and used to calculate relative packaging efficiencies (ii). A genome packaging defect of the mutant virus is often characterized by the relative loss of certain vRNAs in the virion population. The relative number of total particles can be determined using HA assay (iii). A decreased ratio of infectious particles (PFU) to total particles (measured as hemagglutination units [HAU]), is characteristic for a genome packaging defect. (**C**) Analysis of budding viral particles by electron microscopy and electron tomography. Infected cells are fixed, stained and embedded, and a tilt series of an ultrathin section is recorded using an electron microscope. These images can be used to count the number of vRNPs within single viral particles and to reconstruct a 3D presentation of the packaged genome complex. (**D**) Electrophoretic mobility shift assay (EMSA). Pairs of *in vitro* transcribed vRNAs are synthesized, mixed and analysed for vRNA–vRNA complex formation by native agarose gel electrophoresis. A size shift compared to single-vRNA controls indicates complex formation of the two vRNAs. Interaction sites can be mapped by mutating a putative interaction site in one partner vRNA (e.g. B_mut_). Disruption of the vRNA–vRNA complex due to the mutations can be visualized as a loss of the size shift. (**E**) (7 + 2) competition assay. Cells are co-transfected with seven rescue plasmids encoding different vRNAs and two rescue plasmid variants coding for the missing eighth vRNA. These variants can either be a wild-type and a mutated vRNA or vRNA variants of different IAV strains. The released virus particles are subsequently plaque-purified and subjected to genotyping by sequencing. The preferentially packaged vRNA variant is found in the majority of viral plaques.

To map these proposed terminal packaging signals of each vRNA in detail, artificial genome segments were created in which a GFP reporter gene was flanked by the NCRs plus varying portions of the adjacent coding region of the studied vRNA (Figure 2A). In plasmid-based co-transfection assays, such artificial vRNAs were propagated by the IAV replication machinery and subsequently packaged into virus-like particles (VLPs) in the presence of the seven remaining wild-type vRNAs. Cells were subsequently infected with the released VLPs and a helper virus, and successful packaging events of these artificial vRNAs were detected by counting the number of GFP-positive cells. Such (7 + 1) VLP assays were performed with artificial vRNAs derived from all genome segments, allowing the systematic probing of the minimal terminal sequences required for vRNA packaging (46–55). While the exact nucleotide sequences varied depending on the genome segment under study, the NCRs and a minimum of 9–222 nucleotides of the adjacent 3′ and 5′ coding regions were necessary to efficiently package the reporter vRNAs into VLPs (8,56) (Figure 1A). These sequences were similar to those found in DI-RNAs (9), supporting the idea that segment-specific terminal packaging signals drive the incorporation of vRNAs.

**Figure 3.**
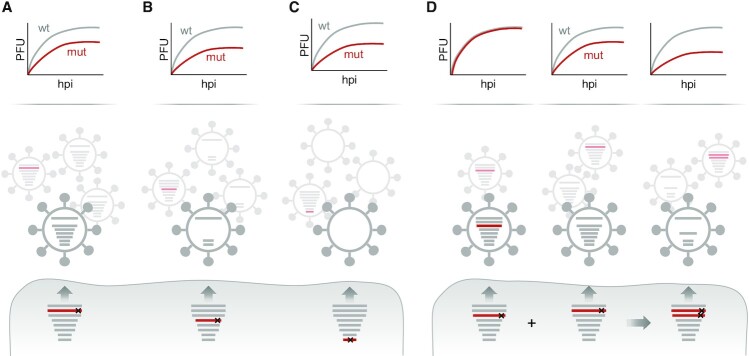
Packaging phenotypes of various IAV packaging mutants. Indicated are schematic growth kinetics (upper panel) and packaging phenotypes (middle panel) of IAVs with mutated packaging signals (mut) compared to a wild-type virus (wt). (**A–C**) Mutations within a packaging signal of one genome segment may cause reduced packaging of the respective vRNA (A), or multiple vRNAs (B), or all vRNAs (C) into the viral particle population resulting in reduced viral growth. (**D**) In some cases, mutations within a packaging signal of one genome segment do not obviously affect genome packaging and viral growth when compared to the wild-type virus. However, combination with another mutated (and thus packaging-defective) vRNA can provoke reduced packaging of several genome segments accompanied by impaired viral growth. PFU, plaque-forming units; hpi, hours post infection.

After the discovery of terminal packaging signals, research was intensified to understand their mechanism of action. Since terminal packaging signals contain conserved nucleotide stretches, a series of WSN/H1N1 and PR8/H1N1 mutants were created, by altering either a single 3′ or 5′ terminal packaging signal of a given genome segment with synonymous nucleotide substitutions (48,57–65). These mutant viruses were then propagated in cell culture to assess the production of infectious particles and packaging of the eight vRNAs (Figure 2B). Intriguingly, many mutant viruses formed more non-infectious particles than a wild-type control virus in compensation for less infectious virions (58,60,62,63,65). While some mutants inefficiently packaged the mutated vRNA (57,59,61,63) (Figure 3A), many other mutants showed impaired packaging of multiple genome segments, which often, but not always, included the mutated one (57,59,60,62–65) (Figure 3B). In rare cases, mutants produced increased amounts of empty virions (65) (Figure 3C) or showed a ‘random’ packaging phenotype characterized by inefficient packaging of all eight vRNAs and the production of vast amounts of non-infectious virions (58). This range of different genome packaging defects suggested that the terminal packaging signals are involved in intricate vRNP–vRNP interactions that coordinate packaging of a genome complex.

Since 3′ and 5′ terminal packaging signals are present in all genome segments, it was envisioned that they collectively participate in a network of vRNP–vRNP interactions involving all vRNPs. Recently, Bolte and colleagues provided compelling evidence for this idea (63). Intrigued by the finding that single mutated terminal packaging signals in vRNAs 1, 2 or 3 provoked none or only minor genome packaging defects in SC35M/H7N7 (as opposed to the same mutations in WSN/H1N1 (59)), they combined these seemingly ‘silent’ mutations to create SC35M mutants with up to three mutated terminal packaging signals (Figure 3D). This approach revealed that the combination of two or three mutated terminal packaging signals caused the formation of non-infectious virions and reduced packaging of multiple vRNAs unlike the single mutations, suggesting that terminal packaging signals are involved in a redundant network of vRNP–vRNP interactions that tolerates the loss of critical vRNP–vRNP contacts to some extent. Interestingly, the packaging phenotypes resulting from different combinations of mutated terminal packaging signals were generally unpredictable, albeit following certain patterns (63). This finding hinted at plastic rearrangements in the network of vRNP–vRNP interactions in response to the loss of certain interactions, potentially mediated by functionally redundant terminal packaging signals.

This apparent flexibility of the vRNP–vRNP interaction network is further supported by findings that mutated terminal packaging signals have varying effects on genome packaging depending on the analysed IAV strain (59,63). Thus, it is plausible that different IAVs use strain-specific vRNP–vRNP interaction networks which respond differently to the same mutation. Although speculative, a flexible rewiring of the interaction network might be achieved through specific combinations of conserved terminal packaging signals in vRNAs 1, 2, 3, 5 and 7 and subtype- or even strain-specific terminal packaging signals in vRNAs 4, 6 and 8 (48,51,66,67).

### The mechanism of action of the terminal packaging signals remains unknown

Although numerous terminal packaging signals were identified, their mechanism of action has remained under investigation ever since. As they consist of RNA nucleotides, it was speculated that they form intersegmental RNA–RNA interactions. Early hints supporting this idea came from electron-tomography experiments that visualized electron-dense structures between neighbouring vRNPs in the (7 + 1) genome complex of budding viral particles (Figure 2C). Fournier and colleagues observed a ‘platform’ located at the top of this complex where the viral polymerases of the vRNPs are presumably located (36). Its size was sufficient to accommodate potential interactions between terminal packaging signals in the vicinity of the viral polymerases. In addition, Noda and colleagues found string-like structures that connected adjacent vRNPs all along their surface, indicating intersegmental contacts mediated by terminal packaging signals and potential internal vRNA regions (35). While these electron-dense structures might indeed represent RNA–RNA interactions between adjacent vRNPs, it has been a challenge to distinguish true RNA–RNA contacts from background noise due to the limited resolution of electron tomography.

**Figure 4.**
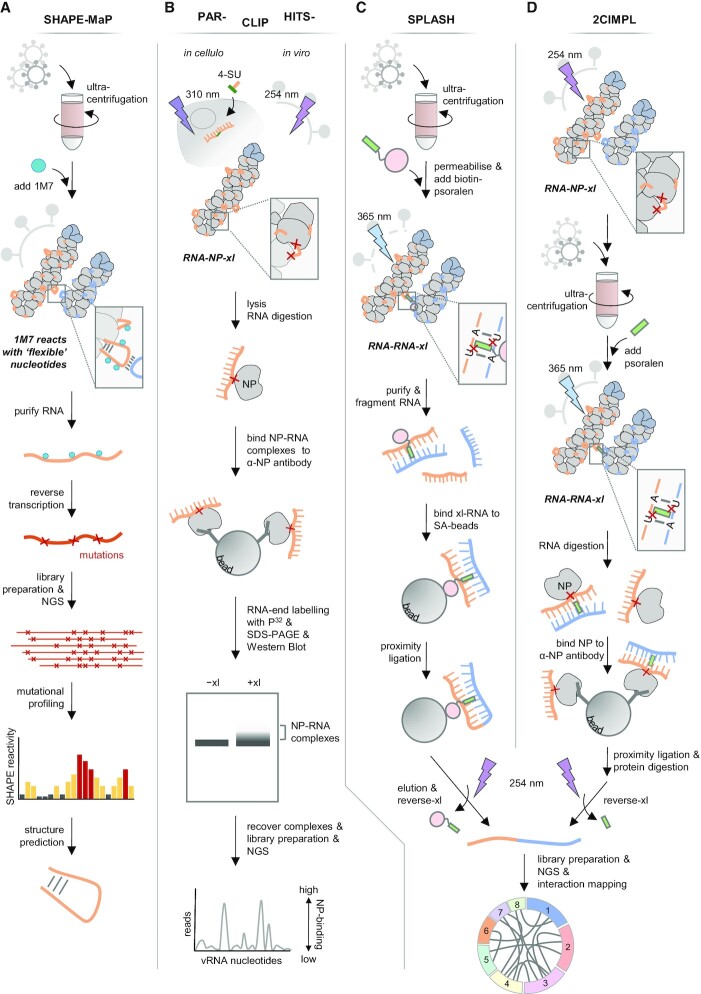
Novel high-throughput methods to study IAV genome packaging. (**A**) Selective 2'-hydroxyl acylation analysed by primer extension and mutational profiling (SHAPE-MaP). Purified viral particles are treated with the SHAPE reagent 1M7 that reacts with the sugar moiety of ‘flexible’ nucleotides. During reverse transcription of the purified RNA, point mutations are introduced at sites modified with 1M7. The resulting cDNA is subjected to library preparation and next generation sequencing (NGS). Mutation frequencies compared to a DMSO-treated control are used to measure SHAPE reactivities and to predict RNA secondary structures. (**B**) Photoactivatable ribonucleoside enhanced-crosslinking immunoprecipitation (PAR-CLIP) and high-throughput sequencing of RNA isolated by crosslinking immunoprecipitation (HITS-CLIP). NP-RNA complexes can be crosslinked in infected cells (‘*in cellulo*’) or virus particles (‘*in viro*’) by using either 4-thiouridin (4-SU) incorporation into newly synthesized RNA followed by UV irradiation at 310 nm or by directly using UV irradiation at 254 nm, respectively. Upon cell/virion lysis, RNA is partially digested, and NP-RNA complexes are enriched using anti-NP antibody coated beads. Radioactive end-labelling of the RNA with P^32^ facilitates visualization of NP-RNA complexes upon SDS-PAGE and Western Blot. NP-RNA complexes are recovered, and the RNA is isolated and subjected to library preparation and NGS. An increase in the normalized read coverage compared to a conventional RNA-seq library of the virus suggests NP-binding sites in the vRNA. (**C**) Sequencing of psoralen crosslinked, ligated, and selected hybrids (SPLASH). Viral particles are ultracentrifuged, permeabilized and treated with biotin-psoralen (biotin, pink circle; psoralen, green rectangle). Upon UV irradiation at 365 nm, psoralen crosslinks (xl), in particular, interstacked pyrimidines. Whole RNA is isolated, fragmented and enriched for biotinylated RNA using streptavidin (SA) beads. Proximity ligation of hybridized and psoralen-bound RNAs leads to the formation of chimeric RNAs. After crosslink-reversal at 254 nm, the RNA is subjected to library preparation followed by NGS. Chimeric RNAs are split-mapped to the IAV genome, and intersegmental RNA–RNA interactions are visualized in circos plots. (**D**) Dual crosslinking, immunoprecipitation, and proximity ligation (2CIMPL). Cell culture supernatant with virus particles is irradiated with 254-nm UV light to crosslink NP-RNA complexes. Virus particles are ultracentrifuged and treated with psoralen and 365-nm UV light to crosslink RNA–RNA interactions. RNA is partially digested, and NP-bound RNA is enriched using anti-NP antibody-coated beads. Proximity ligation of hybridized and NP-bound RNAs leads to the formation of chimeric RNAs. Subsequently, NP is digested and psoralen is removed by UV irradiation at 254 nm. The recovered RNA is subjected to library preparation and NGS. Chimeric RNAs are split-mapped to the IAV genome, and intersegmental RNA–RNA interactions are visualized in circos plots.

The currently favoured mechanistic model proposes that the terminal packaging signals adopt local RNA secondary structure that loops out of the vRNPs and form sequence-specific intermolecular interactions. Such kissing-loop interactions have been previously observed for other viruses where they regulate various processes (68–73). In agreement with this mechanistic model, structural probing of *in vitro* transcribed vRNAs and computational predictions have shown that the terminal packaging signals of several genome segments adopt defined RNA secondary structures (74–80). Moreover, SHAPE-MaP analysis (Figure 4A) of viral particles suggested that some local RNA structures are also present in vRNPs and that the 5′ terminal packaging signals tend to be more structured compared to adjacent internal vRNA regions (81). Finally, CLIP experiments (Figure 4B) indicated that certain parts of the vRNAs, including some terminal packaging signals, are relatively free of NP and thus able to fold into structural elements (19–21).

Some of these identified RNA structure elements were proven crucial for IAV genome packaging. For example, mutational studies confirmed the role of a pseudoknot residing in the 5′ terminal packaging signal of genome segment 5. Disruption of this structural element by mutagenesis caused attenuated viral growth and reduced packaging of multiple genome segments (21,62,74). In contrast, no such genome packaging defect was observed when mutations were designed to preserve or repair the pseudoknot. Similarly, Hagey *et al.* showed the biological significance of a conserved stem-loop within the 5′ terminal packaging signal of genome segment 1 (80). Disruption of this stem-loop either by mutagenesis or treatment with antisense-oligos led to reduced viral infectivity and a drop in the packaging efficiencies of segment 1 and multiple other genome segments. This genome packaging defect could be restored by repairing the stem-loop with compensatory mutations, proving the importance of this structural element. Despite these insights, secondary structures of other terminal packaging signals, especially in vRNPs, and their relevance in genome packaging remain poorly understood.

To identify intersegmental RNA–RNA interactions crucial for IAV genome packaging, Fournier and colleagues transcribed the eight vRNAs of Moscow/H3N2 *in vitro* and analysed their pair-wise interactions in an electrophoretic mobility shift assay (EMSA) (Figure 2D) (36,82). This approach identified numerous *in vitro* RNA–RNA interactions that could be combined into a network comprising all eight vRNAs. Subsequent experiments revealed that some of these *in vitro* interactions were formed by previously defined terminal packaging signals. For example, mutating the 5′ terminal packaging signal of segment 7 largely prevented an *in vitro* interaction between vRNAs 7 and 6. Importantly, these mutations also impaired packaging of the mutated vRNA 7 compared with a wild-type vRNA 7 in a (7 + 2) rescue assay (Figure 2E), suggesting a crucial role of this interaction site. However, the interaction site of vRNA 6 could not be pinpointed, as neither deleting its 3′ nor 5′ packaging signal disrupted the *in vitr*o interaction, leaving the intermolecular interaction and its functional relevance elusive.

Further evidence that terminal packaging signals form intersegmental RNA–RNA interactions was provided by Essere and colleagues while studying reassortment events between Moscow/H3N2 and Finch/H5N2 viruses using a co-transfection-based rescue assay (83). They observed that vRNA 4 of the H5N2 virus was incorporated into the H3N2 genetic background only when combined with the H5N2-vRNA 7. The authors speculated that this co-segregation event was enabled by an RNA–RNA interaction between the H5N2-vRNAs 4 and 7 mediated by terminal packaging signals. Indeed, replacing the 3′ terminal packaging signal of the H3N2-vRNA 7 with that of the H5N2-vRNA 7 was sufficient to facilitate the co-segregation event. However, while the nucleotides of vRNA 7 involved in this putative interaction were mapped in detail, the partner region in vRNA 4 was not identified, which left the exact intersegmental RNA–RNA contact obscure.

Recently, Miyamoto and colleagues described a functional interplay between the terminal packaging signals of vRNAs 1 and 4 in WSN/H1N1 (65). By introducing synonymous mutations into the 5′ terminal packaging signal of vRNA 4 they created a mutant virus that failed to efficiently package vRNAs 4 and 6. Passaging of this packaging mutant in cell culture, however, selected a virus revertant with a wild-type-like packaging phenotype, as it acquired single point mutations in the 5′ terminal packaging signals of genome segments 4 and 1. Although this finding suggested that these modified terminal packaging signals re-established an intersegmental RNA–RNA interaction to alleviate the genome packaging defect, this hypothesis was not unambiguously supported by computational predictions and EMSAs.

In conclusion, the experimental data reviewed thus far suggest a crucial role of the terminal packaging signals in the formation of an octameric genome complex and its incorporation into the viral particle. For some genome segments, the terminal packaging signals are known to adopt RNA secondary structures responsible for coordinated genome packaging. Although a few studies using virus mutants suggest that terminal packaging signals establish RNA–RNA interactions between genome segments, this mechanistic concept remains to be conclusively proven.

### Functional RNA–RNA interactions between internal vRNA regions are known but rare

While many studies have characterized terminal packaging signals, relatively little research has explored a potential role of internal vRNA regions in IAV genome packaging. Nevertheless, a few studies assessed the impact of internal vRNA deletions on virus replication and genome packaging (45,84–86). Recombinant viruses harbouring previously defined DI-vRNAs were generated and propagated in cells *trans*-complemented for the missing viral protein. Subsequent analyses of viral particle preparations by plaque assay (on the *trans*-complemented cells), HA-assay and RT-qPCR (Figure 2B) evaluated whether these clonal DI-viruses had growth and genome packaging defects. Characterization of a virus with a DI-vRNA 1 revealed that this truncated genome segment was inefficiently packaged into viral particles compared to vRNAs 5 and 8 (the other vRNAs were not tested) (45). Moreover, this DI-virus showed a reduced PFU-to-HAU ratio compared to the wild-type control virus, indicating that it formed non-infectious particles which lacked one or multiple full-length vRNAs (85). Together, these findings suggested that this DI-vRNA 1 lacks internal packaging signals crucial to coordinate genome packaging. Furthermore, a recent study found that DI-RNAs derived from genome segments 2, 3 and 4 are also inefficiently packaged into viral particles when compared to their full-length counterparts (43), suggesting the presence of internal packaging signals in these vRNAs. However, whether these DI-RNAs lower the packaging efficiencies of other full-length vRNAs like the tested DI-vRNA 1 remains to be investigated.

Although these deletion studies implied the existence of internal packaging signals, they could not dismiss the possibility that the internal vRNA deletions disturbed the correct folding and functioning of adjacent terminal packaging signals, thereby indirectly causing the observed genome packaging defects. Bolte and colleagues ruled out this ambiguity and identified a putative internal packaging signal by introducing synonymous mutations into a short conserved internal vRNA region of genome segment 3 in SC35M/H7N7 (63). While the exclusive mutation of this region did not provoke a genome packaging defect, combining it with mutations in the 5′ terminal packaging signal of vRNA 2 reduced packaging of four genome segments. Importantly, the virus harbouring only the latter mutations failed to exclusively package vRNA 2. This finding suggested that internal packaging signals indeed exist and contribute together with terminal packaging signals to the vRNP–vRNP interaction network.

While the molecular role of terminal packaging signals in IAV genome packaging remains disputable, a few internal vRNA regions have been proven to form intersegmental RNA–RNA interactions. Gavazzi and colleagues obtained first indications for this concept while investigating pair-wise interactions between *in vitro* transcribed vRNAs of Finch/H5N2 using an EMSA (87,88). They revealed that the eight vRNAs formed a complete intersegmental network that was reminiscent of the network obtained with Moscow/H3N2-derived vRNAs (82). However, in contrast to the *in vitro* interactions of the H3N2-vRNAs that mainly involved terminal packaging signals, multiple *in vitro* interactions of the H5N2-vRNAs formed between internal vRNA regions. Deletion studies and computational predictions allowed the researchers to localize potential interaction sites in the vRNAs that could be later confirmed by disrupting the *in vitro* interactions using antisense-oligos. Importantly, two of these *in vitro* interactions were further validated using *trans*-complementary mutagenesis. In this approach, mutations introduced into either interaction partner disrupted the *in vitro* RNA–RNA interaction, whereas combining both mutated vRNAs restored it, proving that these vRNA regions establish specific intermolecular base-pairings. Despite these findings, none of the *in vitro* RNA–RNA interactions were validated to play a role in IAV genome packaging.

**Figure 5.**
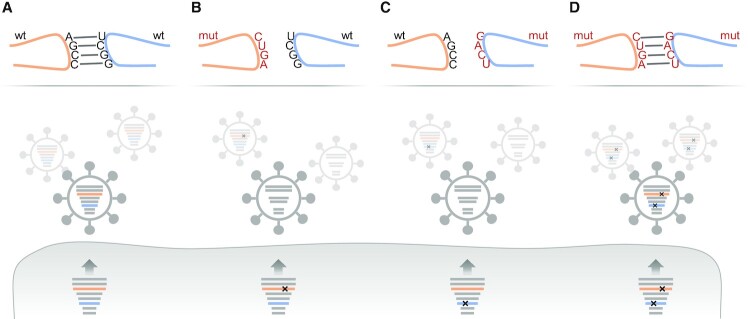
*Trans*-complementary virus mutants as tools to validate vRNA–vRNA interactions important for genome packaging. (**A**) In a wild-type virus, two vRNAs (in orange and blue) form an intersegmental RNA–RNA interaction that is required for genome packaging. (**B**, **C**) Introduction of synonymous mutations into either of the interaction partners abrogates this vRNA–vRNA interaction leading to impaired packaging of the mutated genome segments and possibly other vRNAs. (**D**) Combining the two mutated vRNAs from panels B and C repairs the intersegmental RNA–RNA interaction and restores genome packaging.

However, Gavazzi and colleagues identified in a subsequent study an additional *in vitro* RNA–RNA interaction between internal regions of vRNAs 2 and 8 of Finch/H5N2 (88) that was crucial to IAV genome packaging. By generating *trans*-complementary virus mutants (Figure 5) they could show that mutation of either interaction site decreased the PFU-to-HAU ratio compared to that of the wild-type virus, indicating formation of more non-infectious particles. RT-qPCR experiments revealed that the mutant viruses poorly packaged four vRNAs (the other four vRNAs were not tested), while EM analysis of budding virus particles showed large amounts of empty virions, suggesting that loss of the intersegmental RNA–RNA interaction reduced packaging of all eight vRNAs. Finally, the virus harbouring both mutated genome segments showed a restored PFU-to-HAU ratio and a reduced number of empty virions compared to the single-vRNA mutant viruses, proving that an intermolecular kissing interaction between the two genome segments is crucial for genome packaging. While this intersegmental RNA–RNA contact is important for genome packaging in Finch/H5N2, it may not be relevant to many other IAV strains as sequence analyses suggest that it is only partially conserved in other H5N2 strains and not conserved in other subtypes.

More evidence of intersegmental RNA–RNA interactions involving internal packaging signals was provided by studies investigating reassortment events during IAV vaccine production. IAV vaccines are usually produced in eggs by co-infection of an egg-adapted parental virus (e.g. PR8/H1N1) and a human isolate (e.g. Udorn/H3N2). The resulting reassortant viruses ideally replicate well in eggs and possess vRNAs 4 and 6 of the seasonal virus to elicit an immune response against the surface glycoproteins hemagglutinin and neuraminidase. While analysing reassortant viruses produced from co-infections between PR8 and Udorn, Cobbin and colleagues observed that the Udorn-vRNA 6 often co-segregated with the Udorn-vRNA 2 into the PR8 genetic background but rarely with the PR8-vRNA 2 (89). This imbalance, however, was surprising since both recombinant reassortants replicated efficiently in eggs. Subsequent (7 + 2) competition assays confirmed the preferential co-segregation event of the Udorn-vRNAs 6 and 2 in cell culture and revealed that it depended on a 300-nucleotide spanning region in Udorn-vRNA 2, which lies beyond the terminal packaging signals (90). As described in detail in the following section, a subsequent analysis could pinpoint the exact nucleotides that establish this functional interaction between Udorn-vRNAs 2 and 6 (81).

In conclusion, two intersegmental RNA–RNA interactions between internal vRNA regions could be precisely mapped and validated to coordinate genome packaging. However, the short list of internal packaging signals, in contrast to the extensive list of terminal packaging signals, remains a challenge in evaluating their general role in genome packaging.

### High-throughput probing of vRNA–vRNA interaction networks

Techniques coupling RNA–RNA crosslinking to next generation sequencing have recently enabled the high-throughput identification of intersegmental RNA–RNA interactions. These techniques commonly use psoralen derivates which intercalate into double-stranded RNA regions and crosslink them upon UV irradiation. Ligation of the crosslinked RNA regions creates chimeric RNAs, which are subsequently reverse-transcribed and sequenced. Computational analysis of the chimeric reads recovers the initially crosslinked RNA–RNA interactions, which can be used to build up an interaction network with precise intermolecular base-pairings.

Dadonaite *et al.* performed sequencing of psoralen crosslinked, ligated, and selected hybrids (SPLASH) (Figure 4C) on purified viral particles of WSN/H1N1 (81). They identified an extensive, complex and redundant intersegmental RNA–RNA interaction network comprising hundreds of interactions connecting all eight vRNAs. Importantly, the interaction sites were not restricted to the terminal vRNA regions but distributed along the entire length of the genome segments. In that way, the contacts were either formed between terminal packaging signals, or between internal vRNA regions, or between both. However, the previously described terminal packaging signals showed varying detection frequencies, and many of them were even absent from the 50 most frequent RNA–RNA contacts of the network. Comparative analyses revealed that the SPLASH interaction networks of the closely related WSN/H1N1 and PR8/H1N1 strains were similar, sharing many interactions, albeit the detected frequencies of many overlapping interactions varied. In contrast, the SPLASH network of the distantly related Udorn/H3N2 strain was largely different from these H1N1 networks, sharing only very few contacts, suggesting that specific nucleotide stretches in the vRNAs determine the architecture of the SPLASH networks. In a similar approach, Le Sage *et al.* performed dual crosslinking, immunoprecipitation and proximity ligation (2CIMPL) (Figure 4D) using viral particles of WSN/H1N1 (91). While the 2CIMPL workflow also used psoralen, it implemented some changes compared to SPLASH, one of which was that it mapped RNA–RNA interactions forming between vRNA regions crosslinked to NP. The network identified by 2CIMPL also showed a complex and redundant architecture like the SPLASH network; however, despite using the same virus, only 10% of the identified intersegmental RNA–RNA interactions overlapped. This discrepancy could be due to the different workflows.

The SPLASH and 2CIMPL workflows identified hundreds of novel potential intersegmental RNA–RNA interactions; however, only few of these were assessed for their relevance in IAV genome packaging. Nevertheless, Dadonaite and colleagues could show in a (7 + 2) competition assay that a preferential co-segregation of Udorn-vRNAs 2 and 6 is mediated by specific base pairings between these two genome segments (81). They also confirmed that this interaction occurs in some other H3N2 viruses but is absent in Wyoming/H3N2 due to four nucleotide changes in the interacting site of vRNA 6. Changing these Wyoming-specific nucleotides to the Udorn-specific ones restored the interaction between the Wyoming-vRNAs 2 and 6 and allowed their preferential co-packaging. Importantly, SPLASH analysis of the respective reassortant viruses confirmed the absence or presence of this interaction. In addition, Le Sage *et al.* focused on a ‘hotspot’ region in vRNA 5 that interacted with multiple partner sites on different genome segments (91). Its mutation caused a genome-wide rearrangement of the intersegmental 2CIMPL network. Though this rearrangement was not accompanied by a detectable genome packaging defect, a potential compensatory function of newly established interactions was not addressed. Thus, albeit hundreds of novel intersegmental RNA–RNA interactions were discovered by SPLASH and 2CIMPL, their significance for genome packaging remains largely unknown.

### The prevailing genome packaging model currently fails the stress test

**Figure 6.**
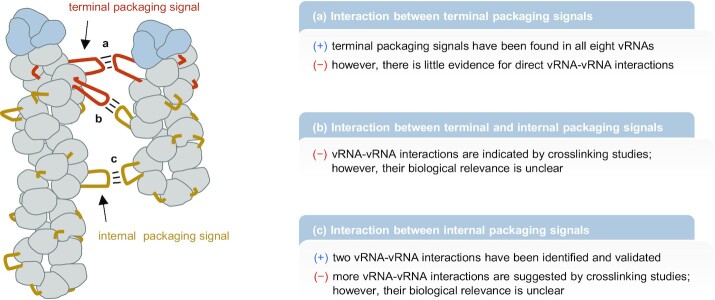
Intersegmental RNA–RNA interactions in IAV genome packaging: an appealing model with open questions. For details see main text.

The eight IAV genome segments are known to be selectively packaged into viral particles as an octameric genome complex. Several lines of evidence support that this process is facilitated by an extensive and partially flexible network of intersegmental RNA–RNA interactions formed by terminal and internal packaging signals. However, two key aspects of this mechanistic model still lack conclusive evidence. Firstly, it has not yet been proven that the terminal packaging signals coordinate genome packaging by forming intersegmental RNA–RNA interactions (Figure 6). Although mutational studies and the SPLASH and 2CIMPL networks suggest that intersegmental RNA–RNA interactions involving terminal packaging signals exist, their relevance in genome packaging remains to be functionally validated. Likewise, only two intersegmental RNA–RNA interactions formed by internal vRNA regions were functionally proven so far (81,88,90). Since these RNA–RNA contacts are virus-strain specific, it is questionable whether interactions involving internal vRNA regions play a major role in genome packaging. Secondly, the relative contribution of terminal and internal packaging signals in genome packaging is currently unclear. While there is an extensive list of packaging mutants harbouring dysfunctional terminal packaging signals, relatively few packaging mutants with mutated internal vRNA regions are known. Nevertheless, it is possible that many more internal packaging signals exist in the IAV genome, and it is tempting to speculate that previous studies have overlooked them by focusing on the terminal vRNA regions due to their presence in DIs and their conservation across IAV strains. Recent studies showing that DI-RNAs are packaged less efficiently than their full-length counterparts indeed suggest the presence of internal packaging signals in many genome segments and pave the way for future studies to identify them.

## TOWARDS A ROBUST GENOME PACKAGING MODEL: PITFALLS AND PROSPECTS

Despite recent progress, our current mechanistic understanding of the genome packaging process is not sufficient to fully accept the prevailing genome packaging model. To develop a robust understanding of the genome packaging mechanism we (i) propose avenues to test the biological significance of the experimentally postulated intersegmental RNA–RNA interaction networks, (ii) suggest to evaluate and improve the accuracy of the existent RNA–RNA interaction probing strategies and (iii) encourage the field to explore the potential role of the IAV nucleoprotein (NP) in modulating intersegmental RNA–RNA contacts.

### Validation of proposed vRNA–vRNA interactions

One of the current challenges in IAV genome packaging is the shortage of validated intersegmental RNA–RNA interactions. An attractive avenue out of this problem might be to extend functional testing of interactions identified by SPLASH and 2CIMPL using *trans*-complementary virus mutants (Figure 5). Thus, the significance of a proposed intersegmental RNA–RNA interaction could be confirmed by showing that its disruption has negative impact on viral growth and genome packaging. Conversely, repairing the targeted interaction through *trans*-complementation would alleviate these defects and prove the base-pairing mechanism.

On the downside, this approach may be challenging. One issue could be that the disruption of a proposed RNA–RNA contact does not lead to a detectable genome packaging defect due to compensatory mechanisms such as mutation-induced global network rearrangements (91) or the presence of functionally redundant interactions (63). In these cases, it might be difficult to prove that the disrupted interaction is nonetheless crucial for genome packaging. While (7 + 2) competition assays may help reveal the impact of the disrupted interaction (81), combinatorial mutagenesis to disrupt the networks at multiple parts could also provide a solution to prove functional importance (63). Another inherent problem of *trans*-complementary mutagenesis is the limited range of available mutations. The nucleotide substitutions must not only be chosen to disrupt the interaction from both sites but also complement each other. In addition, the mutations should ideally be synonymous to preserve the function of the encoded viral proteins. Consequently, some interactions might not be readily confirmable as has been already previously noted (87). Nevertheless, prediction programs can help in the design of suitable *trans*-complementary mutants (92). It is conceivable that this rigorous validation process will prove to be a Sisyphean task and yet, it is a promising option to substantiate the prevailing genome packaging model.

### Finding accurate probing strategies

Apart from the complications discussed above, another problem in the validation process could be false-positive and false-negative RNA–RNA interactions. Mapping of RNA–RNA contacts using EMSAs with *in vitro* transcribed vRNAs were mostly performed in the absence of NP. However, inside viral particles and infected cells, vRNA is bound by NP which influences RNA secondary structure (81,93) and thus possibly also the formation of intersegmental RNA–RNA interactions. Consequently, EMSAs neglecting NP may miss crucial RNA–RNA contacts or identify non-functional ones. Furthermore, EMSAs have only analysed RNA–RNA interactions between pairs of genome segments so far. This artificial situation does not necessarily recapitulate RNA–RNA contacts between eight vRNPs and thus might allow RNA–RNA interactions that are precluded in the genome complex due to specific positioning of the vRNPs (35,36). These methodological problems could be reasons why only one of the many *in vitro* RNA–RNA interactions identified by EMSAs was found crucial in genome packaging.

The discovery of the SPLASH and 2CIMPL networks holds promise of identifying functionally relevant RNA–RNA interactions on a global scale. However, researchers should not assume that these networks represent the true RNA–RNA interaction networks coordinating genome packaging until proven. Indeed, psoralen-based identification workflows tend to introduce specific biases which possibly affect the finally recovered networks. For example, psoralen largely prefers to crosslink RNA–RNA interactions comprising staggered pyrimidines (94). Thus, other RNA–RNA contacts lacking this specific nucleotide composition and geometry are probably absent from the identified networks. Moreover, heavily crosslinked RNA–RNA interactions tend to be lost during the RNA purification procedures used by SPLASH and 2CIMPL (95). This bias could result in a spurious underrepresentation of heavily crosslinked interactions in the obtained networks compared to sparsely crosslinked ones. Another problem might be ‘pseudo-interactions’ that form after the initial crosslinking step at later stages of the workflow through hybridization of single-stranded RNA regions, followed by ligation and detection. Though such ‘pseudo-interactions’ have not been demonstrated so far, the current workflows are not designed to exclude them or control for them. Ultimately, a combination of these and other biases (96) might skew the identified networks far away from the real ones. This might explain why only 10% of the SPLASH and 2CIMPL networks overlap and why these networks lack many of the previously characterized terminal packaging signals.

Such skewed networks would impose a mammoth task on researchers trying to validate interaction candidates by mutagenesis. Determined by the specific workflow, many packaging-relevant RNA–RNA interactions might be masked by a collection of ‘pseudo-interactions’. Likewise, the redundancy and thus mutational robustness of the true interaction network could be underestimated if many packaging-relevant interactions are missed because they are not crosslinked by psoralen. Together, these obstacles may complicate the identification of functional intersegmental RNA–RNA interactions.

Thus, to identify packaging-relevant RNA–RNA interactions, improved or even new strategies might have to be envisioned. While some biases of the SPLASH and 2CIMPL workflows are potentially eliminable (95), others such as the crosslinking preference of psoralen are not. Other probing techniques such as vRIC-seq (97) could be alternatives for the identification of RNA–RNA interactions; however, it is important to note that all currently available probing techniques probably have inherent biases, and therefore cannot draw an accurate picture of the real interaction network on their own. Nevertheless, comparing datasets obtained by multiple probing techniques across related virus strains using suitable statistical frameworks might help identify an overlapping set of candidate interactions that could play a conserved role in genome packaging. In addition, comparative analyses between wild-type viruses and IAVs with mutated terminal packaging signals may offer a shortcut for identifying packaging-relevant RNA–RNA contacts involving terminal vRNA regions.

Besides the technical limitations stated above, the biggest hurdle yet might be to discover the optimal probing material allowing the identification of packaging-relevant RNA–RNA interactions. In the current SPLASH and 2CIMPL workflows, RNA–RNA contacts are probed inside viral particles that have been released from infected cells and subsequently concentrated by ultracentrifugation. This strategy assumes that the intersegmental RNA–RNA interactions crucial for genome packaging are preserved under these conditions. However, it is documented that ultracentrifugation deforms viral particles (98) and possibly rearranges the genome complex. These structural rearrangements might be accompanied by the disruption of essential RNA–RNA interactions or the formation of artificial contacts which would contribute to a skewed interaction network. A better probing material could be budding virus particles because they contain well-organized (7 + 1) genome complexes wherein adjacent vRNPs interact via string-like structures that potentially represent packaging-relevant RNA–RNA interactions (35,36). However, if these contacts are preserved in released viral particles remains disputable as virions shrink after being released from cells (34,35,99), which may induce ‘bending’ of the longest vRNPs and subsequent rearrangements of the genome complex (100). Finally, infected cells could be used to probe intersegmental RNA–RNA interactions. Ideal probing environments could be liquid organelles that form in the cytoplasm during the late phase of infection and probably host IAV genome assembly (32). Though attractive, probing of RNA–RNA interactions inside confined environments such as liquid organelles or budding viral particles would require new sophisticated techniques.

In conclusion, the identification of functional intersegmental RNA–RNA contacts will greatly depend on the accuracy of the applied probing strategies, and mutational analyses will be an important tool to benchmark them. Improvements of the existent probing techniques and development of novel strategies could finally help paint a clear picture of the intersegmental RNA–RNA networks that control genome packaging and reassortment.

### Exploring the potential role of NP in modulating vRNA–vRNA interactions

Only recently, it was recognized that in addition to terminal and internal packaging signals, NP also serves a critical role in genome packaging. NP is the main protein component of vRNPs and consists of a head domain, a body domain, and a flexible tail loop (101). During genome replication, multiple NP molecules oligomerize on the nascent vRNA by inserting the tail loop into an insertion pocket in the body domain of another NP. This NP-vRNA complex folds back and twists around itself to form a helical vRNP together with the viral polymerase (Figure 1B) (23,24,26). Although the details of the vRNP structure are poorly understood, NP likely binds the negatively charged sugar-phosphate backbone of the vRNA through a positively charged RNA-binding groove located between the NP head and body domains, thereby presenting the bases of the bound vRNA outward of the vRNP (93,101–103).

By mutagenesis, Moreira and colleagues identified conserved amino acid residues in the NP head and body domains crucial for genome packaging (104). In their approach, they generated SC35M/H7N7 viruses, in which either seven NP-head domain residues (rNP7) or 18 NP-body domain residues (rCH2) were replaced with the corresponding ones of a distantly related bat-born IAV of the H17N10 subtype. Viral growth and RT-qPCR analyses revealed that these viruses with NP amino acid substitutions produced many non-infectious virions due to reduced packaging of multiple vRNAs. In this way, these NP mutant viruses were reminiscent of SC35M viruses with multiple mutated terminal packaging signals (63), suggesting that both types of alterations impaired the same underlying mechanism. Importantly, the poorly packaged vRNA subsets varied between the NP mutant viruses, indicating that each set of amino acid substitutions disrupted a distinct set of vRNP–vRNP interactions. Additionally, Moreira and colleagues discovered an rNP7-R31G virus revertant with an extra amino acid substitution in the NP-body domain which showed wild-type-like genome packaging (104), suggesting repaired vRNP–vRNP contacts. However, Bolte and colleagues found that adding single mutated terminal packaging signals to the rNP7-R31G genetic background reduced packaging of multiple vRNAs, whereas adding them to the wild-type SC35M genetic background had little or no effect on genome packaging (63), indicating that the rNP7-R31G revertant virus established a distinct vRNP–vRNP interaction network.

These findings have established a crucial role of NP in selective genome packaging; however, the underlying molecular mechanism remains speculative. An attractive scenario is that binding of NP to the vRNAs helps them to adopt their native structure which is crucial to expose packaging signals and establish intersegmental RNA–RNA interactions. This modulatory role of NP is supported by two observations: firstly, SHAPE analyses suggested that although the vRNA structure is mainly determined by its sequence, NP can induce some local structural changes upon binding to the vRNA (81). Secondly, CLIP studies found that the eight vRNAs are non-uniformly bound by NP and retain unbound regions (19–21). Taking these findings together, it is plausible that NP binds to specific vRNA regions and thereby allows neighbouring regions such as packaging signals to remain free and adopt local secondary structures to participate in intersegmental RNA–RNA interactions.

While there is only very limited structural information available on NP-RNA interactions (26,103), multiple amino acid residues within the putative RNA-binding groove of NP have been functionally mapped (101,105–109). Interestingly, some of the rNP7 amino acid residues identified by Moreira and colleagues are identical with or located close to these putative RNA-binding residues (104). Consequently, it is possible that their replacement alters the affinity of NP towards specific vRNA regions and induces vRNA structural changes that impede crucial intersegmental RNA–RNA interactions. This is further supported by the observation that alanine substitutions of basic amino acid residues in the putative RNA-binding groove impair genome packaging (110). Likewise, some of the amino acid residues altered in the rCH2 mutant are located inside or in proximity to an accessory RNA-binding region of NP (104,108) and thus might similarly alter specific vRNA structures and intersegmental RNA–RNA contacts.

Some amino acid residues identified to be important for genome packaging do not lie within known RNA-binding regions of NP (104), suggesting that they are not directly involved in RNA binding. It is possible that these NP amino acid residues influence vRNA structuring through NP–NP interactions that control the relative positioning of NP molecules and their RNA-binding regions within vRNPs. Since RNA-binding is distributed across multiple NP molecules in vRNPs, the overall configuration of the NP backbone might be involved in structuring the bound vRNAs in their entirety. While early cryo-EM studies revealed a rigid configuration of the NP backbone showing a regular helical structure (23,24), recent cryo-EM studies identified the NP backbone to be structurally flexible and contain NP molecules with distinct orientations (25,26). These flexible NP orientations may place RNA-binding regions at specific positions in the NP backbone, thereby guiding which vRNA regions are bound by NP and helping the encapsidated vRNA to find its native structure that exposes packaging signals for intersegmental RNA–RNA interactions. Consequently, certain NP amino acid substitutions could disrupt essential intersegmental RNA–RNA contacts by changing the NP backbone configuration through altered NP–NP interactions.

These mechanistic possibilities have yet to be explored, and it will be critical in the future to test whether NP mutant viruses, such as those found by Moreira and colleagues, display alterations in vRNA structure and intersegmental RNA–RNA interactions responsible for the observed genome packaging defects. The success of these studies will depend on accurate techniques to probe intersegmental RNA–RNA interactions as discussed in the preceding sections. However, understanding how NP possibly modulates vRNA–vRNA interactions will require a broader panel of sophisticated and accurate techniques that is suitable to decipher additional changes in RNA–NP and NP–NP interactions as well as changes in the vRNP configuration between wild-type and NP mutant viruses.

## CONCLUDING REMARKS

Here, we provided a comprehensive description of known packaging signals in the IAV genome segments and challenged the prevailing mechanistic model that they establish a specific, yet flexible network of intersegmental RNA–RNA interactions. This mechanistic model emerged with the discovery of the terminal packaging signals that provided an intuitive explanation for how mutually interacting genome segments could be packaged into virus particles in the form of a supramolecular complex. Eventually, the discovery of two functional RNA–RNA interactions between internal vRNA regions has provided a proof of this mechanistic concept. However, the lack of functional vRNA–vRNA interactions formed by terminal packaging signals remains a major weak point that needs to be addressed in the future.

While crosslinking-based RNA–RNA interaction probing techniques hold promise of identifying additional functional vRNA–vRNA contacts, it becomes evident that they might suffer from biases that portray a distorted image of the vRNA–vRNA interaction networks coordinating IAV genome packaging. Nevertheless, careful consideration of these biases and improved experimental designs coupled with *trans*-complementary mutagenesis may eventually expand the limited set of validated intersegmental RNA–RNA contacts and clarify the roles played by terminal packaging signals and internal regions in IAV genome packaging.

The recent discovery that NP is involved in IAV genome packaging suggests an additional level of mechanistic complexity that awaits future investigation. While the precise role of NP is currently unclear, NP mutant viruses with genome packaging defects could serve as valuable tools to identify functional RNA–RNA interactions involved in IAV genome packaging.

Finally, we would like to mention that (i) the roles played by other viral proteins and host cell factors in IAV genome packaging and (ii) a systematic analysis of the current bioinformatics approaches to predict and study the involved intersegmental RNA–RNA interactions lie beyond the scope of this review and therefore have not been reviewed here. Understandably, insights obtained on these aspects could contribute significantly to our current knowledge of IAV genome packaging and genetic reassortment.

## References

[1] Taubenberger J.K. , Kash J.C. Influenza virus evolution, host adaptation, and pandemic formation. Cell Host Microbe. 2010; 7:440–451. 20542248 10.1016/j.chom.2010.05.009 PMC2892379

[2] Kessler S. , Harder T.C., Schwemmle M., Ciminski K. Influenza A viruses and zoonotic events-are we creating our own reservoirs?. Viruses. 2021; 13:2250. 34835056 10.3390/v13112250 PMC8624301

[3] Smith G.J. , Vijaykrishna D., Bahl J., Lycett S.J., Worobey M., Pybus O.G., Ma S.K., Cheung C.L., Raghwani J., Bhatt S. et al. Origins and evolutionary genomics of the 2009 swine-origin H1N1 influenza A epidemic. Nature. 2009; 459:1122–1125. 19516283 10.1038/nature08182

[4] Viboud C. , Simonsen L., Fuentes R., Flores J., Miller M.A., Chowell G. Global mortality impact of the 1957–1959 influenza pandemic. J. Infect. Dis. 2016; 213:738–745. 26908781 10.1093/infdis/jiv534 PMC4747626

[5] Honigsbaum M. Revisiting the 1957 and 1968 influenza pandemics. Lancet. 2020; 395:1824–1826. 32464113 10.1016/S0140-6736(20)31201-0 PMC7247790

[6] Henritzi D. , Petric P.P., Lewis N.S., Graaf A., Pessia A., Starick E., Breithaupt A., Strebelow G., Luttermann C., Parker L.M.K. et al. Surveillance of european domestic pig populations identifies an emerging reservoir of potentially zoonotic swine influenza A viruses. Cell Host Microbe. 2020; 28:614–627. 32721380 10.1016/j.chom.2020.07.006

[7] Sun H. , Xiao Y., Liu J., Wang D., Li F., Wang C., Li C., Zhu J., Song J., Sun H. et al. Prevalent eurasian avian-like H1N1 swine influenza virus with 2009 pandemic viral genes facilitating human infection. Proc. Natl. Acad. Sci. U.S.A. 2020; 117:17204–17210. 32601207 10.1073/pnas.1921186117 PMC7382246

[8] Gerber M. , Isel C., Moules V., Marquet R. Selective packaging of the influenza A genome and consequences for genetic reassortment. Trends Microbiol. 2014; 22:446–455. 24798745 10.1016/j.tim.2014.04.001

[9] Hutchinson E.C. , von Kirchbach J.C., Gog J.R., Digard P. Genome packaging in influenza A virus. J. Gen. Virol. 2010; 91:313–328. 19955561 10.1099/vir.0.017608-0

[10] Lowen A.C. Constraints, drivers, and implications of influenza A virus reassortment. Annu Rev Virol. 2017; 4:105–121. 28548881 10.1146/annurev-virology-101416-041726

[11] Ferhadian D. , Contrant M., Printz-Schweigert A., Smyth R.P., Paillart J.C., Marquet R. Structural and functional motifs in influenza virus RNAs. Front Microbiol. 2018; 9:559. 29651275 10.3389/fmicb.2018.00559 PMC5884886

[12] Zhao L. , Peng Y., Zhou K., Cao M., Wang J., Wang X., Jiang T., Deng T. New insights into the nonconserved noncoding region of the subtype-determinant hemagglutinin and neuraminidase segments of influenza A viruses. J. Virol. 2014; 88:11493–11503. 25056889 10.1128/JVI.01337-14 PMC4178829

[13] Benkaroun J. , Robertson G.J., Whitney H., Lang A.S. Analysis of the variability in the non-coding regions of influenza A viruses. Vet Sci. 2018; 5:76. 10.3390/vetsci5030076 PMC6165000 30149635

[14] Bae S.H. , Cheong H.K., Lee J.H., Cheong C., Kainosho M., Choi B.S. Structural features of an influenza virus promoter and their implications for viral RNA synthesis. Proc. Natl. Acad. Sci. U.S.A. 2001; 98:10602–10607. 11553808 10.1073/pnas.191268798 PMC58512

[15] Park C.J. , Bae S.H., Lee M.K., Varani G., Choi B.S. Solution structure of the influenza A virus cRNA promoter: implications for differential recognition of viral promoter structures by RNA-dependent RNA polymerase. Nucleic Acids Res. 2003; 31:2824–2832. 12771209 10.1093/nar/gkg387 PMC156722

[16] Pflug A. , Guilligay D., Reich S., Cusack S. Structure of influenza A polymerase bound to the viral RNA promoter. Nature. 2014; 516:355–360. 25409142 10.1038/nature14008

[17] Fan H. , Walker A.P., Carrique L., Keown J.R., Serna Martin I., Karia D., Sharps J., Hengrung N., Pardon E., Steyaert J. et al. Structures of influenza A virus RNA polymerase offer insight into viral genome replication. Nature. 2019; 573:287–290. 31485076 10.1038/s41586-019-1530-7 PMC6795553

[18] Fodor E. , Te Velthuis A.J.W. Structure and function of the influenza virus transcription and replication machinery. Cold Spring Harb. Perspect. Med. 2020; 10:a038398. 31871230 10.1101/cshperspect.a038398 PMC7334866

[19] Lee N. , Le Sage V., Nanni A.V., Snyder D.J., Cooper V.S., Lakdawala S.S. Genome-wide analysis of influenza viral RNA and nucleoprotein association. Nucleic Acids Res. 2017; 45:8968–8977. 28911100 10.1093/nar/gkx584 PMC5587783

[20] Le Sage V. , Nanni A.V., Bhagwat A.R., Snyder D.J., Cooper V.S., Lakdawala S.S., Lee N Non-uniform and non-random binding of nucleoprotein to influenza A and B viral RNA. Viruses. 2018; 10:522. 10.3390/v10100522 PMC6213415 30257455

[21] Williams G.D. , Townsend D., Wylie K.M., Kim P.J., Amarasinghe G.K., Kutluay S.B., Boon A.C.M. Nucleotide resolution mapping of influenza A virus nucleoprotein-RNA interactions reveals RNA features required for replication. Nat. Commun. 2018; 9:465. 29386621 10.1038/s41467-018-02886-w PMC5792457

[22] Compans R.W. , Content J., Duesberg P.H. Structure of the ribonucleoprotein of influenza virus. J. Virol. 1972; 10:795–800. 4117350 10.1128/jvi.10.4.795-800.1972 PMC356535

[23] Arranz R. , Coloma R., Chichon F.J., Conesa J.J., Carrascosa J.L., Valpuesta J.M., Ortin J., Martin-Benito J. The structure of native influenza virion ribonucleoproteins. Science. 2012; 338:1634–1637. 23180776 10.1126/science.1228172

[24] Moeller A. , Kirchdoerfer R.N., Potter C.S., Carragher B., Wilson I.A. Organization of the influenza virus replication machinery. Science. 2012; 338:1631–1634. 23180774 10.1126/science.1227270 PMC3578580

[25] Gallagher J.R. , Torian U., McCraw D.M., Harris A.K. Structural studies of influenza virus RNPs by electron microscopy indicate molecular contortions within NP supra-structures. J. Struct. Biol. 2017; 197:294–307. 28007449 10.1016/j.jsb.2016.12.007 PMC5360478

[26] Coloma R. , Arranz R., de la Rosa-Trevín J.M., Sorzano C.O.S., Munier S., Carlero D., Naffakh N., Ortín J., Martín-Benito J. Structural insights into influenza A virus ribonucleoproteins reveal a processive helical track as transcription mechanism. Nat. Microbiol. 2020; 5:727–734. 32152587 10.1038/s41564-020-0675-3

[27] Chou Y.Y. , Heaton N.S., Gao Q., Palese P., Singer R.H., Lionnet T. Colocalization of different influenza viral RNA segments in the cytoplasm before viral budding as shown by single-molecule sensitivity FISH analysis. PLoS Pathog. 2013; 9:e1003358. 23671419 10.1371/journal.ppat.1003358 PMC3649991

[28] Lakdawala S.S. , Wu Y., Wawrzusin P., Kabat J., Broadbent A.J., Lamirande E.W., Fodor E., Altan-Bonnet N., Shroff H., Subbarao K. Influenza A virus assembly intermediates fuse in the cytoplasm. PLoS Pathog. 2014; 10:e1003971. 24603687 10.1371/journal.ppat.1003971 PMC3946384

[29] Giese S. , Bolte H., Schwemmle M. The feat of packaging eight unique genome segments. Viruses. 2016; 8:165. 10.3390/v8060165 PMC4926185 27322310

[30] de Castro Martin I.F. , Fournier G., Sachse M., Pizarro-Cerda J., Risco C., Naffakh N. Influenza virus genome reaches the plasma membrane via a modified endoplasmic reticulum and Rab11-dependent vesicles. Nat. Commun. 2017; 8:1396. 29123131 10.1038/s41467-017-01557-6 PMC5680169

[31] Vale-Costa S. , Amorim M.J. Clustering of rab11 vesicles in influenza A virus infected cells creates hotspots containing the 8 viral ribonucleoproteins. Small GTPases. 2017; 8:71–77. 27337591 10.1080/21541248.2016.1199190 PMC5464114

[32] Alenquer M. , Vale-Costa S., Etibor T.A., Ferreira F., Sousa A.L., Amorim M.J. Influenza A virus ribonucleoproteins form liquid organelles at endoplasmic reticulum exit sites. Nat. Commun. 2019; 10:1629. 30967547 10.1038/s41467-019-09549-4 PMC6456594

[33] Haralampiev I. , Prisner S., Nitzan M., Schade M., Jolmes F., Schreiber M., Loidolt-Krüger M., Jongen K., Chamiolo J., Nilson N. et al. Selective flexible packaging pathways of the segmented genome of influenza A virus. Nat. Commun. 2020; 11:4355. 32859915 10.1038/s41467-020-18108-1 PMC7455735

[34] Noda T. , Sagara H., Yen A., Takada A., Kida H., Cheng R.H., Kawaoka Y. Architecture of ribonucleoprotein complexes in influenza A virus particles. Nature. 2006; 439:490–492. 16437116 10.1038/nature04378

[35] Noda T. , Sugita Y., Aoyama K., Hirase A., Kawakami E., Miyazawa A., Sagara H., Kawaoka Y. Three-dimensional analysis of ribonucleoprotein complexes in influenza A virus. Nat. Commun. 2012; 3:639. 22273677 10.1038/ncomms1647 PMC3272569

[36] Fournier E. , Moules V., Essere B., Paillart J.C., Sirbat J.D., Isel C., Cavalier A., Rolland J.P., Thomas D., Lina B. et al. A supramolecular assembly formed by influenza A virus genomic RNA segments. Nucleic Acids Res. 2012; 40:2197–2209. 22075989 10.1093/nar/gkr985 PMC3300030

[37] Nakatsu S. , Sagara H., Sakai-Tagawa Y., Sugaya N., Noda T., Kawaoka Y. Complete and incomplete genome packaging of influenza A and B viruses. MBio. 2016; 7:e01248-16. 27601575 10.1128/mBio.01248-16 PMC5013298

[38] Jennings P.A. , Finch J.T., Winter G., Robertson J.S. Does the higher order structure of the influenza virus ribonucleoprotein guide sequence rearrangements in influenza viral RNA?. Cell. 1983; 34:619–627. 6616623 10.1016/0092-8674(83)90394-x

[39] Dimmock N.J. , Easton A.J. Defective interfering influenza virus RNAs: time to reevaluate their clinical potential as broad-spectrum antivirals?. J. Virol. 2014; 88:5217–5227. 24574404 10.1128/JVI.03193-13 PMC4019098

[40] Nayak D.P. , Chambers T.M., Akkina R.K. Defective-interfering (DI) RNAs of influenza viruses: origin, structure, expression, and interference. Curr. Top. Microbiol. Immunol. 1985; 114:103–151. 3888540 10.1007/978-3-642-70227-3_3

[41] Pelz L. , Rüdiger D., Dogra T., Alnaji F.G., Genzel Y., Brooke C.B., Kupke S.Y., Reichl U. Semi-continuous propagation of influenza A virus and its defective interfering particles: analyzing the dynamic competition to select candidates for antiviral therapy. J. Virol. 2021; 95:e0117421. 34550771 10.1128/JVI.01174-21 PMC8610589

[42] Mendes M. , Russell A.B. Library-based analysis reveals segment and length dependent characteristics of defective influenza genomes. PLoS Pathog. 2021; 17:e1010125. 34882752 10.1371/journal.ppat.1010125 PMC8691639

[43] Alnaji F.G. , Reiser W.K., Rivera-Cardona J., Te Velthuis A.J.W., Brooke C.B. Influenza A virus defective viral genomes are inefficiently packaged into virions relative to wild-type genomic RNAs. Mbio. 2021; 12:e0295921. 34809454 10.1128/mBio.02959-21 PMC8609359

[44] Huang A.S. , Baltimore D. Defective viral particles and viral disease processes. Nature. 1970; 226:325–327. 5439728 10.1038/226325a0

[45] Hein M.D. , Arora P., Marichal-Gallardo P., Winkler M., Genzel Y., Pöhlmann S., Schughart K., Kupke S.Y., Reichl U. Cell culture-based production and in vivo characterization of purely clonal defective interfering influenza virus particles. BMC Biol. 2021; 19:91. 33941189 10.1186/s12915-021-01020-5 PMC8091782

[46] Fujii Y. , Goto H., Watanabe T., Yoshida T., Kawaoka Y. Selective incorporation of influenza virus RNA segments into virions. Proc. Natl. Acad. Sci. U.S.A. 2003; 100:2002–2007. 12574509 10.1073/pnas.0437772100 PMC149948

[47] Watanabe T. , Watanabe S., Noda T., Fujii Y., Kawaoka Y. Exploitation of nucleic acid packaging signals to generate a novel influenza virus-based vector stably expressing two foreign genes. J. Virol. 2003; 77:10575–10583. 12970442 10.1128/JVI.77.19.10575-10583.2003 PMC228515

[48] Fujii K. , Fujii Y., Noda T., Muramoto Y., Watanabe T., Takada A., Goto H., Horimoto T., Kawaoka Y. Importance of both the coding and the segment-specific noncoding regions of the influenza A virus NS segment for its efficient incorporation into virions. J. Virol. 2005; 79:3766–3774. 15731270 10.1128/JVI.79.6.3766-3774.2005 PMC1075679

[49] Liang Y. , Hong Y., Parslow T.G. cis-Acting packaging signals in the influenza virus PB1, PB2, and PA genomic RNA segments. J. Virol. 2005; 79:10348–10355. 16051827 10.1128/JVI.79.16.10348-10355.2005 PMC1182667

[50] Muramoto Y. , Takada A., Fujii K., Noda T., Iwatsuki-Horimoto K., Watanabe S., Horimoto T., Kida H., Kawaoka Y. Hierarchy among viral RNA (vRNA) segments in their role in vRNA incorporation into influenza A virions. J. Virol. 2006; 80:2318–2325. 16474138 10.1128/JVI.80.5.2318-2325.2006 PMC1395381

[51] Gog J.R. , Afonso Edos S., Dalton R.M., Leclercq I., Tiley L., Elton D., von Kirchbach J.C., Naffakh N., Escriou N., Digard P. Codon conservation in the influenza A virus genome defines RNA packaging signals. Nucleic Acids Res. 2007; 35:1897–1907. 17332012 10.1093/nar/gkm087 PMC1874621

[52] Ozawa M. , Fujii K., Muramoto Y., Yamada S., Yamayoshi S., Takada A., Goto H., Horimoto T., Kawaoka Y. Contributions of two nuclear localization signals of influenza A virus nucleoprotein to viral replication. J. Virol. 2007; 81:30–41. 17050598 10.1128/JVI.01434-06 PMC1797272

[53] Liang Y. , Huang T., Ly H., Parslow T.G., Liang Y. Mutational analyses of packaging signals in influenza virus PA, PB1, and PB2 genomic RNA segments. J. Virol. 2008; 82:229–236. 17959657 10.1128/JVI.01541-07 PMC2224372

[54] Ozawa M. , Maeda J., Iwatsuki-Horimoto K., Watanabe S., Goto H., Horimoto T., Kawaoka Y. Nucleotide sequence requirements at the 5' end of the influenza A virus m RNA segment for efficient virus replication. J. Virol. 2009; 83:3384–3388. 19158245 10.1128/JVI.02513-08 PMC2655591

[55] Goto H. , Muramoto Y., Noda T., Kawaoka Y. The genome-packaging signal of the influenza A virus genome comprises a genome incorporation signal and a genome-bundling signal. J. Virol. 2013; 87:11316–11322. 23926345 10.1128/JVI.01301-13 PMC3807325

[56] Li X. , Gu M., Zheng Q., Gao R., Liu X. Packaging signal of influenza A virus. Virol J. 2021; 18:36. 33596956 10.1186/s12985-021-01504-4 PMC7890907

[57] Marsh G.A. , Hatami R., Palese P. Specific residues of the influenza A virus hemagglutinin viral RNA are important for efficient packaging into budding virions. J. Virol. 2007; 81:9727–9736. 17634232 10.1128/JVI.01144-07 PMC2045411

[58] Hutchinson E.C. , Curran M.D., Read E.K., Gog J.R., Digard P. Mutational analysis of cis-acting RNA signals in segment 7 of influenza A virus. J. Virol. 2008; 82:11869–11879. 18815307 10.1128/JVI.01634-08 PMC2583641

[59] Marsh G.A. , Rabadan R., Levine A.J., Palese P. Highly conserved regions of influenza A virus polymerase gene segments are critical for efficient viral RNA packaging. J. Virol. 2008; 82:2295–2304. 18094182 10.1128/JVI.02267-07 PMC2258914

[60] Hutchinson E.C. , Wise H.M., Kudryavtseva K., Curran M.D., Digard P. Characterisation of influenza A viruses with mutations in segment 5 packaging signals. Vaccine. 2009; 27:6270–6275. 19840659 10.1016/j.vaccine.2009.05.053 PMC2771075

[61] Wise H.M. , Barbezange C., Jagger B.W., Dalton R.M., Gog J.R., Curran M.D., Taubenberger J.K., Anderson E.C., Digard P. Overlapping signals for translational regulation and packaging of influenza A virus segment 2. Nucleic Acids Res. 2011; 39:7775–7790. 21693560 10.1093/nar/gkr487 PMC3177217

[62] Anhlan D. , Hrincius E.R., Scholtissek C., Ludwig S. Introduction of silent mutations into the NP gene of influenza A viruses as a possible strategy for the creation of a live attenuated vaccine. Vaccine. 2012; 30:4480–4489. 22575164 10.1016/j.vaccine.2012.04.070

[63] Bolte H. , Rosu M.E., Hagelauer E., Garcia-Sastre A., Schwemmle M. Packaging of the influenza virus genome is governed by a plastic network of RNA- and Nucleoprotein-Mediated interactions. J. Virol. 2019; 93:e01861-18. 30463968 10.1128/JVI.01861-18 PMC6363987

[64] Seshimo E. , Momose F., Morikawa Y. Identification of the 5'-Terminal packaging signal of the H1N1 influenza A virus neuraminidase segment at single-nucleotide resolution. Front Microbiol. 2021; 12:709010. 34456891 10.3389/fmicb.2021.709010 PMC8385638

[65] Miyamoto S. , Muramoto Y., Shindo K., Fujita-Fujiharu Y., Morikawa T., Tamura R., Gilmore J.L., Nakano M., Noda T. Contribution of RNA–RNA interactions mediated by the genome packaging signals for the selective genome packaging of influenza A virus. J. Virol. 2022; 96:e01641-21. 10.1128/jvi.01641-21 PMC8941900 35044211

[66] White M.C. , Steel J., Lowen A.C. Heterologous packaging signals on segment 4, but not segment 6 or segment 8, limit influenza A virus reassortment. J. Virol. 2017; 91:e00195-17. 28331085 10.1128/JVI.00195-17 PMC5432880

[67] White M.C. , Tao H., Steel J., Lowen A.C. H5N8 and H7N9 packaging signals constrain HA reassortment with a seasonal H3N2 influenza A virus. Proc. Natl. Acad. Sci. U.S.A. 2019; 116:4611–4618. 30760600 10.1073/pnas.1818494116 PMC6410869

[68] Paillart J.C. , Skripkin E., Ehresmann B., Ehresmann C., Marquet R. A loop-loop “kissing” complex is the essential part of the dimer linkage of genomic HIV-1 RNA. Proc. Natl. Acad. Sci. U.S.A. 1996; 93:5572–5577. 8643617 10.1073/pnas.93.11.5572 PMC39288

[69] Sit T.L. , Vaewhongs A.A., Lommel S.A. RNA-mediated trans-activation of transcription from a viral RNA. Science. 1998; 281:829–832. 9694655 10.1126/science.281.5378.829

[70] Lindenbach B.D. , Sgro J.Y., Ahlquist P. Long-distance base pairing in flock house virus RNA1 regulates subgenomic RNA3 synthesis and RNA2 replication. J. Virol. 2002; 76:3905–3919. 11907230 10.1128/JVI.76.8.3905-3919.2002 PMC136111

[71] Friebe P. , Boudet J., Simorre J.P., Bartenschlager R. Kissing-loop interaction in the 3' end of the hepatitis c virus genome essential for RNA replication. J. Virol. 2005; 79:380–392. 15596831 10.1128/JVI.79.1.380-392.2005 PMC538730

[72] Tsetsarkin K.A. , Liu G., Shen K., Pletnev A.G. Kissing-loop interaction between 5' and 3' ends of tick-borne langat virus genome ‘bridges the gap’ between mosquito- and tick-borne flaviviruses in mechanisms of viral RNA cyclization: applications for virus attenuation and vaccine development. Nucleic Acids Res. 2016; 44:3330–3350. 26850640 10.1093/nar/gkw061 PMC4838367

[73] Imperatore J.A. , Cunningham C.L., Pellegrene K.A., Brinson R.G., Marino J.P., Evanseck J.D., Mihailescu M.R. Highly conserved s2m element of SARS-CoV-2 dimerizes via a kissing complex and interacts with host miRNA-1307-3p. Nucleic Acids Res. 2022; 50:1017–1032. 34908151 10.1093/nar/gkab1226 PMC8789046

[74] Gultyaev A.P. , Tsyganov-Bodounov A., Spronken M.I., van der Kooij S., Fouchier R.A., Olsthoorn R.C. RNA structural constraints in the evolution of the influenza A virus genome NP segment. RNA Biol. 2014; 11:942–952. 25180940 10.4161/rna.29730 PMC4179967

[75] Gultyaev A.P. , Spronken M.I., Richard M., Schrauwen E.J., Olsthoorn R.C., Fouchier R.A. Subtype-specific structural constraints in the evolution of influenza A virus hemagglutinin genes. Sci. Rep. 2016; 6:38892. 27966593 10.1038/srep38892 PMC5155281

[76] Michalak P. , Soszynska-Jozwiak M., Biala E., Moss W.N., Kesy J., Szutkowska B., Lenartowicz E., Kierzek R., Kierzek E. Secondary structure of the segment 5 genomic RNA of influenza A virus and its application for designing antisense oligonucleotides. Sci. Rep. 2019; 9:3801. 30846846 10.1038/s41598-019-40443-7 PMC6406010

[77] Lenartowicz E. , Kesy J., Ruszkowska A., Soszynska-Jozwiak M., Michalak P., Moss W.N., Turner D.H., Kierzek R., Kierzek E. Self-folding of naked segment 8 genomic RNA of influenza A virus. PLoS One. 2016; 11:e0148281. 26848969 10.1371/journal.pone.0148281 PMC4743857

[78] Ruszkowska A. , Lenartowicz E., Moss W.N., Kierzek R., Kierzek E. Secondary structure model of the naked segment 7 influenza A virus genomic RNA. Biochem. J. 2016; 473:4327–4348. 27694388 10.1042/BCJ20160651

[79] Soszynska-Jozwiak M. , Pszczola M., Piasecka J., Peterson J.M., Moss W.N., Taras-Goslinska K., Kierzek R., Kierzek E. Universal and strain specific structure features of segment 8 genomic RNA of influenza A virus-application of 4-thiouridine photocrosslinking. J. Biol. Chem. 2021; 297:101245. 34688660 10.1016/j.jbc.2021.101245 PMC8666676

[80] Hagey R.J. , Elazar M., Tian S., Pham E.A., Kladwang W., Ben-Avi L., Nguyen K., Xiong A., Rabinovich M., Schaffert S. et al. Identification and targeting of a pan-genotypic influenza A virus RNA structure that mediates packaging and disease. 2021; bioRxiv doi:21 August 2021, preprint: not peer reviewed 10.1101/2021.08.21.457170.

[81] Dadonaite B. , Gilbertson B., Knight M.L., Trifkovic S., Rockman S., Laederach A., Brown L.E., Fodor E., Bauer D.L.V. The structure of the influenza A virus genome. Nat Microbiol. 2019; 4:1781–1789. 31332385 10.1038/s41564-019-0513-7 PMC7191640

[82] Fournier E. , Moules V., Essere B., Paillart J.C., Sirbat J.D., Cavalier A., Rolland J.P., Thomas D., Lina B., Isel C. et al. Interaction network linking the human H3N2 influenza A virus genomic RNA segments. Vaccine. 2012; 30:7359–7367. 23063835 10.1016/j.vaccine.2012.09.079

[83] Essere B. , Yver M., Gavazzi C., Terrier O., Isel C., Fournier E., Giroux F., Textoris J., Julien T., Socratous C. et al. Critical role of segment-specific packaging signals in genetic reassortment of influenza A viruses. Proc. Natl. Acad. Sci. U.S.A. 2013; 110:E3840–E3848. 24043788 10.1073/pnas.1308649110 PMC3791739

[84] de Wit E. , Spronken M.I., Rimmelzwaan G.F., Osterhaus A.D., Fouchier R.A. Evidence for specific packaging of the influenza A virus genome from conditionally defective virus particles lacking a polymerase gene. Vaccine. 2006; 24:6647–6650. 16831497 10.1016/j.vaccine.2006.06.001

[85] Yamagata Y. , Muramoto Y., Miyamoto S., Shindo K., Nakano M., Noda T. Generation of a purely clonal defective interfering influenza virus. Microbiol. Immunol. 2019; 63:164–171. 30997933 10.1111/1348-0421.12681

[86] Bdeir N. , Arora P., Gartner S., Hoffmann M., Reichl U., Pohlmann S., Winkler M. A system for production of defective interfering particles in the absence of infectious influenza A virus. PLoS One. 2019; 14:e0212757. 30822349 10.1371/journal.pone.0212757 PMC6396908

[87] Gavazzi C. , Isel C., Fournier E., Moules V., Cavalier A., Thomas D., Lina B., Marquet R. An in vitro network of intermolecular interactions between viral RNA segments of an avian H5N2 influenza A virus: comparison with a human H3N2 virus. Nucleic Acids Res. 2013; 41:1241–1254. 23221636 10.1093/nar/gks1181 PMC3553942

[88] Gavazzi C. , Yver M., Isel C., Smyth R.P., Rosa-Calatrava M., Lina B., Moules V., Marquet R. A functional sequence-specific interaction between influenza A virus genomic RNA segments. Proc. Natl. Acad. Sci. U.S.A. 2013; 110:16604–16609. 24067651 10.1073/pnas.1314419110 PMC3799358

[89] Cobbin J.C. , Ong C., Verity E., Gilbertson B.P., Rockman S.P., Brown L.E. Influenza virus PB1 and neuraminidase gene segments can cosegregate during vaccine reassortment driven by interactions in the PB1 coding region. J. Virol. 2014; 88:8971–8980. 24872588 10.1128/JVI.01022-14 PMC4136297

[90] Gilbertson B. , Zheng T., Gerber M., Printz-Schweigert A., Ong C., Marquet R., Isel C., Rockman S., Brown L. Influenza NA and PB1 gene segments interact during the formation of viral progeny: localization of the binding region within the PB1 gene. Viruses. 2016; 8:238. 10.3390/v8080238 PMC4997600 27556479

[91] Le Sage V. , Kanarek J.P., Snyder D.J., Cooper V.S., Lakdawala S.S., Lee N Mapping of influenza virus RNA–RNA interactions reveals a flexible network. Cell Rep. 2020; 31:107823. 32610124 10.1016/j.celrep.2020.107823 PMC7372595

[92] Desiro D. , Holzer M., Ibrahim B., Marz M. SilentMutations (SIM): a tool for analyzing long-range RNA–RNA interactions in viral genomes and structured RNAs. Virus Res. 2019; 260:135–141. 30439394 10.1016/j.virusres.2018.11.005 PMC7172452

[93] Baudin F. , Bach C., Cusack S., Ruigrok R.W. Structure of influenza virus RNP. I. Influenza virus nucleoprotein melts secondary structure in panhandle RNA and exposes the bases to the solvent. EMBO J. 1994; 13:3158–3165. 8039508 10.1002/j.1460-2075.1994.tb06614.x PMC395207

[94] Cimino G.D. , Gamper H.B., Isaacs S.T., Hearst J.E. Psoralens as photoactive probes of nucleic acid structure and function: organic chemistry, photochemistry, and biochemistry. Annu. Rev. Biochem. 1985; 54:1151–1193. 2411210 10.1146/annurev.bi.54.070185.005443

[95] Zhang M. , Li K., Bai J., Velema W.A., Yu C., van Damme R., Lee W.H., Corpuz M.L., Chen J.F., Lu Z. Optimized photochemistry enables efficient analysis of dynamic RNA structuromes and interactomes in genetic and infectious diseases. Nat. Commun. 2021; 12:2344. 33879794 10.1038/s41467-021-22552-y PMC8058046

[96] Zhang M. , Hwang I.T., Li K., Bai J., Chen J.F., Weissman T., Zou J.Y., Lu Z. Classification and clustering of RNA crosslink-ligation data reveal complex structures and homodimers. Genome Res. 2022; 32:968–985. 35332099 10.1101/gr.275979.121 PMC9104705

[97] Cao C. , Cai Z., Xiao X., Rao J., Chen J., Hu N., Yang M., Xing X., Wang Y., Li M. et al. The architecture of the SARS-CoV-2 RNA genome inside virion. Nat. Commun. 2021; 12:3917. 34168138 10.1038/s41467-021-22785-x PMC8225788

[98] Sugita Y. , Noda T., Sagara H., Kawaoka Y. Ultracentrifugation deforms unfixed influenza A virions. J. Gen. Virol. 2011; 92:2485–2493. 21795472 10.1099/vir.0.036715-0 PMC3352361

[99] Harris A. , Cardone G., Winkler D.C., Heymann J.B., Brecher M., White J.M., Steven A.C. Influenza virus pleiomorphy characterized by cryoelectron tomography. Proc. Natl. Acad. Sci. U.S.A. 2006; 103:19123–19127. 17146053 10.1073/pnas.0607614103 PMC1748186

[100] Slater A. , Nair N., Suétt R., Mac Donnchadha R., Bamford C., Jasim S., Livingstone D., Hutchinson E. Visualising viruses. J. Gen. Virol. 2022; 103:001730. 10.1099/jgv.0.001730 PMC8895616 35082014

[101] Ye Q. , Krug R.M., Tao Y.J. The mechanism by which influenza A virus nucleoprotein forms oligomers and binds RNA. Nature. 2006; 444:1078–1082. 17151603 10.1038/nature05379

[102] Klumpp K. , Ruigrok R.W., Baudin F. Roles of the influenza virus polymerase and nucleoprotein in forming a functional RNP structure. EMBO J. 1997; 16:1248–1257. 9135141 10.1093/emboj/16.6.1248 PMC1169723

[103] Tang Y.S. , Xu S., Chen Y.W., Wang J.H., Shaw P.C. Crystal structures of influenza nucleoprotein complexed with nucleic acid provide insights into the mechanism of RNA interaction. Nucleic Acids Res. 2021; 49:4144–4154. 33784403 10.1093/nar/gkab203 PMC8053115

[104] Moreira E.A. , Weber A., Bolte H., Kolesnikova L., Giese S., Lakdawala S., Beer M., Zimmer G., Garcia-Sastre A., Schwemmle M. et al. A conserved influenza A virus nucleoprotein code controls specific viral genome packaging. Nat. Commun. 2016; 7:12861. 27650413 10.1038/ncomms12861 PMC5035998

[105] Kobayashi M. , Toyoda T., Adyshev D.M., Azuma Y., Ishihama A. Molecular dissection of influenza virus nucleoprotein: deletion mapping of the RNA binding domain. J. Virol. 1994; 68:8433–8436. 7966640 10.1128/jvi.68.12.8433-8436.1994 PMC237318

[106] Albo C. , Valencia A., Portela A. Identification of an RNA binding region within the N-terminal third of the influenza A virus nucleoprotein. J. Virol. 1995; 69:3799–3806. 7745727 10.1128/jvi.69.6.3799-3806.1995 PMC189097

[107] Elton D. , Medcalf L., Bishop K., Harrison D., Digard P. Identification of amino acid residues of influenza virus nucleoprotein essential for RNA binding. J. Virol. 1999; 73:7357–7367. 10438825 10.1128/jvi.73.9.7357-7367.1999 PMC104262

[108] Ng A.K. , Zhang H., Tan K., Li Z., Liu J.H., Chan P.K., Li S.M., Chan W.Y., Au S.W., Joachimiak A. et al. Structure of the influenza virus A H5N1 nucleoprotein: implications for RNA binding, oligomerization, and vaccine design. FASEB J. 2008; 22:3638–3647. 18614582 10.1096/fj.08-112110 PMC2537428

[109] Tarus B. , Chevalier C., Richard C.A., Delmas B., Di Primo C., Slama-Schwok A. Molecular dynamics studies of the nucleoprotein of influenza A virus: role of the protein flexibility in RNA binding. PLoS One. 2012; 7:e30038. 22272272 10.1371/journal.pone.0030038 PMC3260217

[110] Li Z. , Watanabe T., Hatta M., Watanabe S., Nanbo A., Ozawa M., Kakugawa S., Shimojima M., Yamada S., Neumann G. et al. Mutational analysis of conserved amino acids in the influenza A virus nucleoprotein. J. Virol. 2009; 83:4153–4162. 19225007 10.1128/JVI.02642-08 PMC2668439

[111] Isel C. , Munier S., Naffakh N. Experimental approaches to study genome packaging of influenza A viruses. Viruses. 2016; 8:218. 10.3390/v8080218 PMC4997580 27517951

